# Cells of human breast milk

**DOI:** 10.1186/s11658-017-0042-4

**Published:** 2017-07-13

**Authors:** Malgorzata Witkowska-Zimny, Ewa Kaminska-El-Hassan

**Affiliations:** Department of Biophysics and Human Physiology, Medical University of Warsaw, Chalubinskiego 5, 02-004 Warsaw, Poland

**Keywords:** Human breast milk, Stem cells, Leukocytes, Microbiome, Probiotic bacteria

## Abstract

Human milk is a complex fluid that has developed to satisfy the nutritional requirements of infants. In addition to proteins, lipids, carbohydrates and other biologically active components, breast milk contains a diverse microbiome that is presumed to colonize the infant gastrointestinal tract and a heterogeneous population of cells with unclear physiological roles and health implications. Noteworthy cellular components of breast milk include progenitor/stem cells. This review summarizes the current state of knowledge of breast milk cells, including leukocytes, epithelial cells, stem cells and potentially probiotic bacteria.

## Background

Breast milk’s nutritional properties have been recognized for hundreds of years. Breastfeeding is regarded as one of the most important measures to improve children’s health in many societies and breast milk is now considered a therapeutic agent suitable for use in parallel to drug therapy [1–3].

The milk of each species has a unique composition that has evolved over millions of years to suit the needs of infants of that species. It contains a myriad of immunological, biochemical and cellular components that have the potential to significantly alter newborn immunity and susceptibility to infection [1, 4]. Additional complexity is generated by individual variations in breast milk composition, which are attributed to the stage of lactation, the degree of breast fullness, infant feeding, the health of the breastfeeding dyad, and other factors.

**Fig. 1:**
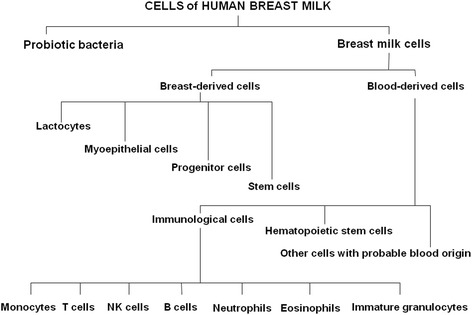
Cells present in human breast milk

Despite variation in milk composition, the main building blocks of milk are common to all mammals. Functionally, it is possible to distinguish between nutritional and bioactive components in mother’s milk. The latter are growth and immunological factors and cellular components. Typically, breast milk is thought to contain epithelial cells and immune cells. Recent breakthroughs have shown that breast milk is more heterogeneous than previously thought and that it also contains stem cells. Furthermore, breast milk is also a continuous source of commensal and beneficial bacteria, including lactic acid bacteria and bifidobacteria. A comparison of somatic cell number and bacterial load in the same samples revealed no significant correlation. The current knowledge of the cellular composition of human milk is summarized in Fig. 1.

Research has shown a close association between milk fat and cell contents that changes with the degree of breast fullness [5]. Mechanisms that remain to be elucidated include the regulation of breast milk synthesis, the migration of cells into breast milk, creation of progenitor/stem cell composition, and creation of the microbiome contribution. The compositional variety of breast milk cell populations raises questions about the function of non-immune and stem/progenitor cells, and the correlations between milk microbiota, somatic cells and macronutrients. This review highlights the current state of knowledge about the cellular composition of human breast milk.

### Immunological cells

Human milk-mediated protection of the infant has long been known and intensively studied. Breast milk confers active and passive immunity to the infant because it is a rich source of immunoglobulins, lactoferrin, lysozymes, cytokines, and numerous other immunological factors.

In the late 1960s, studies revealed that colostrum is rich in leukocytes [6, 7], which were considered the most abundant breast milk cells. However, visual identification results in misidentification and overestimation of leukocyte concentration, whereas new methods like multicolor flow cytometry provide superior identification and quantification of all breast milk cells. New data have revealed that leucocytes constitute only a small minority (<2%) of the cells in the mature milk of a healthy mother [8]. Leukocytes primarily provide active immunity and promote the development of immunocompetence in the infant, but it is also probable that they protect the mammary gland against infection.

The transfer of immune factors from the mother to the infant starts in utero and continues postnatally through breastfeeding [9]. Evidence from animal studies suggest that breast milk leukocytes survive passage through the infant’s digestive tract, and then translocate from the gastrointestinal tract to the blood and distant sites, including the lymph nodes, spleen and liver [10, 11]. However, there are numerous gaps in the knowledge of the development of the immune system and digestive tract in infants. It is known that maternal leukocytes from breast milk provide active immunity to the infant by fighting pathogens directly via phagocytosis, producing bioactive components, assisting in the development of the newborn immune system, or modifying the microenvironment of the infant digestive tract [12]. There are many possibilities for passage through the infant’s digestive tract and translocation from the gastrointestinal tract to the blood (mucosa-associated lymphoid tissues). Breast milk leukocytes have been shown to be activated, motile and interactive, and they can be transferred via the systemic circulation into distant tissues [13]. It has been postulated that miRNAs, which are abundant in breast milk, also participate in leukocyte survival in the infant’s gastrointestinal tract, potentially conferring immunoprotective and developmental functions [14].

The stage of lactation is associated with major changes in milk leukocyte composition [15]. Using multicolor flow cytometry to identify and quantify leukocyte subsets in breast milk obtained from healthy women, Trend et al. found that colostrum contains approximately 146,000 cells/ml and that the amount decreases in transitional (8–12 day postpartum) and mature milk (26–30 day postpartum) to 27,500 and 23,650 cells/ml, respectively [15]. They also demonstrated that breast milk contains a greater variety and complexity of leukocyte subsets than previously thought. Of the identified cells, the major leukocytes present were the myeloid precursors (9–20%), neutrophils (12–27%), immature granulocytes (8–17%), and non-cytotoxic T cells (6–7%). Progression of lactation is associated with decreasing major CD45^+^ leukocyte concentration, eosinophils, myeloid and B cell precursors, and CD16^−^ monocytes. The relative frequencies of neutrophils and immature granulocytes significantly increased in mature milk in comparison to colostrum.

Hassiotou et al. demonstrated a specific increase in breast milk leukocytes when the breastfeeding mother had an infection [8]. Interestingly, Riskin et al. also reported an increase in breast milk leukocytes when the infant has an infection, suggesting a dynamic interaction between the sick babies and their mothers [16]. The dynamic response of breast milk leukocytes to infections indicates that this is a tightly regulated process aimed at conferring additional immunological support to the infant [8, 16]. Further studies are needed to shed light on the immunological mechanisms underlying these responses, as well as their clinical significance.

In addition to blood-derived leukocytes, preliminary studies indicate the presence of hematopoietic stem/progenitor cells in colostrum, which originate from the maternal bloodstream [17]. Their properties, role and mechanism of transfer from maternal blood into breast milk require further study.

### Non-immune cells and stem/progenitor human breast milk cells

While the nutritional and protective function of breast milk has been previously examined, little is known about the properties and roles of the non-immune cells that are present. Studies performed in the 1950s revealed that colostrum contains epithelial cells [18]. In the last decade, it was shown that in addition to these cell populations, breast milk contains stem and progenitor cells [19, 20]. The presence of stem and progenitor cells in the mammary gland and breast milk was postulated earlier based on the ability of the mammary gland to program changes and transforms into the fully secretory state during pregnancy and in the postpartum period.

Thus, human breastmilk contains heterogeneous cell populations including lactocytes (milk-secretory cells), myoepithelial cells (from the ducts and alveoli of mammary gland) and a hierarchy of progenitor and stem cells. The cellular composition of human milk is dynamic and the proportion of different cell types can be changed by many factors, such as stage of lactation, health, and infant feeding. Selected reports on the somatic cells isolated from the breast milk of healthy women are summarized in Table 1.

**Table 1:** Somatic cells content in fresh breast milk when both mother and infant are healthy

Somatic cells	Markers	% of the total cell population		References
		Colostrum (1 day prepartum or 1 day postpartum)	Peak lactation (month postpartum)	
Leukocytes	CD45	13–20	1–2	[15, 54]
Myoepithelial cells	CK5, CK14, CK18, CK19, CD49f, SMA	50–90	60–98	[24, 55]
Lactocytes	CK18, EPCAM			
Breast milk stem cells (hBSCs)	CD44, ITGB1/CD29, ATXN1/SCA1	10–15	No data	[22, 25, 55]
Mesenchymal stem cells (MSCs)	CD90, CD105, CD73, VIM			

**Fig. 2:**
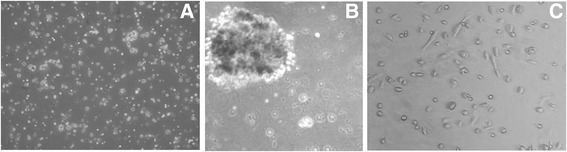
Morphology of the breast milk-derived cells. **a** Heterogeneous cell population including leukocytes. **b** Mammosphere created by hBSCs on Matrigel (on day 8 after isolation). **c** Subpopulation of lactocytes and myoepithelial cells on day 2 after isolation, culture in vitro on tissue culture plates

Luminal and myoepithelial cells and their precursors represent nearly 98% of the non-immune cell types in human milk under healthy conditions. They express a few membrane antigens: CK5, CK14 and CK18, which are markers of differentiation of mammary epithelial cells. Myoepithelial cells build smooth muscle fibers surrounding the alveoli. Their contraction results in the expulsion of milk from the alveoli into the milk ducts. Luminal cells express epithelial cell adhesion molecule (EPCAM), whereas myoepithelial cells express smooth muscle actin (SMA) and cytokeratin 14 (CK14). Lactocytes line the alveoli of the human mammary gland and are responsible for the synthesis and secretion of milk into the alveolar lumen. These alveolar cells express cytokeratin 18 (CK18) and synthesize milk proteins such as α-lactalbumin and ß-casein [21]. Mammary precursors to both luminal and myoepithelial cell types express α6 integrin (CD49f) and cytokeratin 5 (CK5). Many studies demonstrate that epithelial cells isolated from fresh breast milk are adherent cells that form colonies of various morphologies that can be maintained through multiple in vitro culture passages [22, 23]. A similar cell morphology is also observed in our laboratory (Fig. 2).

The presence of nestin, a neuroectoderm marker, is also reported in a subpopulation of breast milk-derived cells. However, the frequency of nestin-positive cells is low in the heterogeneous population of mother’s milk [24].

Cregan et al. showed that breast milk contains cells with stem/progenitor properties [19]. Hosseini et al. found that breast milk-derived stem cells had the capability to differentiate into neural cell lineages and demonstrated their similarity to both embryonic and mesenchymal stem cells. The exposure of the cell population from breast milk to neurogenic medium in vitro led to differentiation into all three neural lineages: neurons expressing ß-tubulin as a neuron marker, oligodendrocytes expressing the O4 marker, and astrocytes expressing the GFAP marker [23]. Both the mammary gland and nervous system have the same embryonic origin, so breastmilk cells could be a good source for neural cell lineage differentiation. It is possible that the cells could be involved in the development of the enteric nervous system, which is one of the main parts of the nervous system, consisting of a mesh-like system of neurons that governs the function of the gastrointestinal system. Non-breast-fed premature born infants show a significantly higher risk of developing diseases like infantile diarrhea and necrotizing enterocolitis.

A few studies suggested that human milk contains mesenchymal stem cells (MSCs). In a study conducted in 2013, cells expressing the typical MSC markers, like CD90, CD105 and CD73, were isolated from breast milk [22, 25]. However, according to Kakulas et al., no convincing evidence currently exists supporting the presence of MSCs in breast milk [26].

The existence of pluripotent stem cells within human breast milk (human breast milk stem cells, hBSCs) was reported for the first time in 2012 by Hassiotou et al. [20]. The authors demonstrated hBSC ability to produce self-renewing stem cells, with a multilineage differentiation potential for all three germ layers: ectoderm, mesoderm and endoderm. They showed the expression of typical embryonic stem cell factors: octamer-binding transcription factor 4 (OCT4), sex determining region Y-box (SOX2), and homeobox (NANOG). They also showed the formation of ESC-like colony morphology and phenotype, but they did not produce teratomas in vivo in immunodeficient mice [27].

Interestingly, a significant upregulation of ESC genes was observed during spheroid formation. It was equal to or sometimes exceeded the expression levels of hESCs. A time-course analysis of OCT4, SOX2 and NANOG mRNA expression from days 1 to 12 of spheroid formation revealed a stable upregulation of these genes.

It has been shown that hBSCs may differentiate in vitro into adipose cells, chondrocytes, osteoblasts, neuronal cells, hepatocyte-like cells and pancreatic beta cells. They are also capable of differentiating into lactocytes and myoepithelial cells. Human breast stem cells can be enriched in suspension cultures as mammospheres. However, little is known about the behavior of these cells. It is possible that hBSCs may be responsible not only for remodeling of the breast necessary to support its development toward a mature milk-secretory organ, but also proliferation, development or epigenetic regulation of tissues in the infant. Studies in mice provide the evidence of migration and integration of breast milk stem cells to organs of the neonate. These cells have been shown to survive and cross the gastrointestinal tract mucosa of nursed mouse pups in vivo, transfer into the bloodstream and further into different organs where they integrate and differentiate into functional cells [28]. This can be an example of human microchimerism. No cells of fetal origin have been observed in the isolates [29].

Very little is known about the milk cells, their origin, properties, and the factors influencing them. It was found that at least some of these cells originate from the mammary epithelium of the lactating breast, but the factors which activate them during pregnancy and lactation are still unknown. It is possible that hBSCs may originate from the maternal bloodstream, like the CD34+ hematopoietic stem cells also present in human milk [17].

Without any doubt, breast milk contains a hierarchy of cells from early-stage embryonic-like stem cells to fully differentiated mammary epithelial cells. Future studies will explore the potential and benefits of non-immune cells and stem/progenitor human breast milk cells in feeding of infants, but also in therapy and regenerative medicine.

### Probiotics: the friendly bacteria in human milk

Human milk is far from being a sterile fluid. The existence of the human milk microbiome was discovered only a decade ago. It is estimated that an infant feeding on 800 ml of breast milk per day could ingest 10^7^–10^8^ bacterial cells daily [30]. Advances in the assessment of early host–microbe interactions suggest that early colonization of the infant gut by milk bacteria may have an impact on disease prevention in children and later health.

The most frequent bacteria found in human milk are those belonging to the species *Staphylococcus*, *Acinetobacter*, *Streptococcus*, *Pseudomonas*, *Lactococcus*, *Enterococcus* and *Lactobacillus* [31]. Some, like *Staphylococcus*, *Corynebacterium* or *Propionibacterium*, can be isolated from the skin and are also frequently found in human milk. They probably prevent from colonization of the host by more severe pathogens, such as *S. aureus* [32]. Others, including *L. gasseri*, *L. salivarius*, *L. rhamnosus*, *L. plantarum* and *L. fermentum,* are considered probiotic species by the European Food Safety Authority (EFSA).

In-depth analysis of the bacterial communities in milk with high-throughput sequencing techniques identified a much greater diversity of bacteria in milk than what previously reported in culture-independent studies that relied on narrower range (quantitative PCR) or precise (PCR-DGGE) methods.

Without a doubt, bacteria are not contamination occurring during sample extraction, as was assumed in past [33–35]. However, the variations may be attributable to genetic, cultural, environmental, or dietary differences among studied populations and human milk microbiome changes during lactation [30, 36]. Interestingly, mother’s milk was found to have similar microbial profiles independently of the age of gestation or mode of delivery [37]. Probiotic bacteria in human milk are a very recent field of research.

Selected reports of the bacterial species isolated from the breast milk of healthy women are summarized Table 2. A few studies suggest that selected bacteria of the maternal gastrointestinal microbiota can access the mammary gland through an entero-mammary pathway. The mechanism involves dendritic cells and CD18^+^ cells, which can take up nonpathogenic bacteria from the gut lumen and carry them to the lactating mammary gland [38, 39]. Boix-Amoros et al. confirmed the presence of live bacteria moving inside the extracellular matrix of immune cells [30]. In another study, bacterial translocation from gut to mesenteric lymph nodes and mammary glands in pregnant and lactating mice was observed [40]. It has been suggested that bacterial translocation to extraintestinal tissues is a beneficial physiological event in a healthy host, and it may be associated with maturation of the neonatal immune system.

**Table 2:** Probiotic bacterial species isolated from the breast milk of healthy women

Bacterial group	Main species	References
*Bifidobacterium*	*B. longum*	[40, 47]
	*B breve*	[47]
	*B lactis*	[47]
	*B. adolescentis*	[33]
*Lactobacillus*	*L. salivarius* CECT5713	[39, 56, 57]
	*L. gasseri* CECT5714	[39, 57]
	*L. plantarum*	[47, 58]
	*L. fermentum* CECT5716	[57, 59]
	*L. rhamnosus*	[47]
	*L. reuteri*	[47]
	*L. acidophilus*	[60]

## Conclusions

During pregnancy, labor and lactation, a gradual remodeling of the mammary gland occurs, facilitated by the orchestrated secretion of the lactogenic hormone complex, which acts on mammary stem and progenitor cells.

Milk composition varies and depend on stage of lactation, the degree of breast fullness, infant feeding, the mother and infant health status, and many other factors and may be associated with the maternal diet and environment, and potentially with genetic factors [41].

Milk is a complex fluid composed of several phases that can be separated by centrifugation into a cream layer, an aqueous phase and a pellet that consists of milk cells. The heterogeneous mixture of breast milk cells includes leukocytes, epithelial cells, stem cells, and bacteria. Certainly, cells of human milk are not an insignificant component, but their function is still unclear. Leukocytes are the most widely studied cell type in breast milk due to their protective properties and their ability to infiltrate the infant’s tissue.

Small non-coding RNAs (miRNAs) are involved in regulation of T- and B-cell development, release of inflammatory mediators, proliferation of neutrophils and monocytes, and the function of dendritic cells and macrophages [42]. Human breast milk is rich in miRNAs and so far, more than 386 different miRNAs were identified in this fluid [43]. The levels of miRNAs and their expression in human milk are lower in colostrum compared to mature milk. The function of extracellular microRNA is still poorly understood, but evidence supports the notion that those RNAs play crucial role in cell-cell communication and besides their role in regulation the immune system, microRNAs might be engaged in the epigenetic regulation of stem cells fate and function.

The discovery of hBSCs with multilineage differentiation potential raised numerous questions concerning the fate of these cells in the infant body and their potential use in regenerative medicine. The breast milk-derived stem cells showed the capacity to be differentiated into neural cell lineages, and their similarity to both embryonic and mesenchymal stem cells makes them a good candidate for cell therapy in neurodegenerative diseases without any ethical concern. hBSCs may be used for autologous cell therapies of the breast milk donor or of individuals having a matching immunogenicity profile. Breast milk stem cells can be also used to improve understanding of the biology of the lactating breast as well as the etiology of lactation difficulties.

Although the mononuclear cells in human milk provide protection, they may also transfer infectious particles from the mother to the infant. RNA retroviruses, including HIV, HTLV-1 and HTLV-2, use this route to infect infants. Other viruses including cytomegalovirus (CMV) and human herpes virus have been identified in human milk, and may be infectious to babies. Viruses may exist freely in breast milk, but are also found within the cells. Maternal milk cells have the potential to act as Trojan horses, carrying viral material into the neonatal gut and lymphoid tissues.

Milk also contains a number of substances that may inhibit viral infection: lactoferrin, antibodies (in particular IgA), and epidermal growth factor prevent the vertical transmission of viruses [44]. However, perinatal guidelines from WHO and European and U.S. authorities state that women with HIV and HTLV should not breastfeed, and instead feed their babies with formula or banked breast milk. Women who are infected with CMV or herpes virus can still breastfeed infants born full-term [45]. A deeper understanding of this fundamental aspect of mammalian biology and the development of some method to block this route of infection requires a concerted approach by scientists, midwives and clinicians.

Probiotic bacteria in human milk contribute to the establishment of the infant microbiome. They can regulate infant immune function and enhance defense against intestinal pathogens. Currently, clinical studies are in progress to evaluate the tolerance and effectiveness of some breast milk strains as a source of potential probiotic bacteria. [46]. The results of Soto et al. confirm that lactobacilli and bifidobacteria are common members of the human milk microbiota of women who did not receive antibiotics during pregnancy or lactation, and the presence of such bacteria may be a marker of a healthy non-antibiotic-altered human milk microbiota, and this should be taken into account when defining a criterion standard for breast milk [47].

Some authors proposed that human milk should be considered as probiotic or even symbiotic food [48]. Jimenez et al. suggested that breast milk can be used as an effective alternative to antibiotics for the treatment of infectious mastitis during lactation [49]. The milk microbiome can influence commensal oral and gut infant bacteria but also their skin microbiota. There are a few reports on the topical application of human milk as an effective treatment for diaper rash, atopic eczema, diaper dermatitis or umbilical cord separation [50–52]. Generally, human milk can be easy, cheap, safe and non-invasive therapeutic approach. However, study with a larger data set is essential to determine the effectiveness of human breast milk in the non-feeding treatments.

Human milk feeding is associated with substantial benefits. Biochemical and cellular components of breast milk are associated with the early life of the infant, conferring not only short-term effects, such as growth, but also long-term benefits, including supporting neurocognitive function, protection against overweight and obesity, hypertension, type 2 diabetes and atopic disease during adolescence and adulthood [44, 53].

Nowadays, we still do not know or understand the relationship between milk microbiota, macronutrients and somatic cell content, and their health implications. Further studies are required to understand the precise nature of breast milk stem/progenitor cells and to explore their potential clinical applications. Considering its composition, function, rich biological ingredients and cellular contents, breast milk can be considered a living tissue.

## Abbreviations

EFSA: European Food Safety Authority
EPCAM: Epithelial cell adhesion molecule
ESCs: Embryonic stem cells
hBSCs: Human breast milk stem cells
MSCs: Mesenchymal stem cells
OCT4: Octamer-binding transcription factor 4
SMA: Smooth muscle actin
SOX2: Sex-determining region Y-box 2
